# Genetic Variances and Heritabilities of Traits of an Early Yellow Maize Population after Cycles of Improvement for Striga Resistance and Drought Tolerance

**DOI:** 10.2135/cropsci2017.10.0628

**Published:** 2018-09-13

**Authors:** B. Badu-Apraku, B. E. Ifie, A. O. Talabi, E. Obeng-Bio, R. Asiedu

**Affiliations:** 1 International Institute of Tropical Agriculture, P.M.B. 5320, Ibadan, Nigeria; 2 West Africa Centre for Crop Improvement, University of Ghana, Legon, Ghana

## Abstract

Drought and *Striga* are principal constraints to maize (*Zea mays* L.) production in sub-Saharan Africa. An early yellow maize population, TZE-Y Pop DT STR, which had undergone five cycles of selection for resistance to *Striga*, followed by three cycles of improvement for drought tolerance, was investigated for yield gains, changes in genetic variance, and interrelationships among traits under drought stress and optimum environments. Two hundred and forty S_1_ lines comprising 60 each from the base population and subsequent populations from three selection cycles improved for grain yield and drought tolerance were assessed under drought and optimal environments in Nigeria from 2010 to 2012. Genetic improvements in grain yield of 423 and 518 kg ha^–1^ cycle^–1^ were achieved under drought stress and optimal environments. Predicted improvements in selection for yield were 348 and 377 kg ha^–1^ cycle^–1^ under drought stress and optimum environments, respectively. The highest yield observed in C_3_ was accompanied by reduced days to silking and anthesis–silking interval, improved plant aspect and ear aspect, and increased plant height and ears per plant across research environments, as well as improved stay-green characteristic under drought. The level of genetic variability for yield and a few other traits were maintained under drought and optimal environments in the population. The presence of residual genetic variability for yield and other assayed traits in C_3_ indicated that progress could be made from future selection in the population depending on the ability of breeders to identify outstanding genotypes and the precision level of experimentation. Substantial improvement has been made in yield and drought tolerance in C_3_ of the population.

Recurrent drought, *Striga hermonthica* (Del.) Benth. infestation, and soil N deficiency are the most prominent limitations to sustainable maize (*Zea mays* L.) production and productivity in West and Central Africa (WCA). Losses in grain yield resulting from *Striga hermonthica* damage on maize could be as high as 100% and force farmers to abandon their farms. About 40 million ha of cereal fields in WCA alone are seriously infested by *Striga* spp., whereas at least 70 million ha have moderate levels of infestation by the parasite (Lagoke et al., 1991). Edmeades et al. (1995) observed that 15% of annual maize yield loss is caused by drought stress in the West African savannas. The authors concluded that yield losses could be greater in the marginal rainfall areas characterized by annual rainfall of <500 mm and sandy or shallow soils. Greater yield losses could be recorded if drought occurred at the most drought-sensitive stages of crop growth and development, particularly during flowering and grain-filling periods (Denmead and Shaw, 1960; NeSmith and Ritchie, 1992). Annually, ~10 to 50% of yield reduction in maize is attributed to stress due to low N (Wolfe et al., 1988). Therefore, the development and genetic enhancement of maize for tolerance to low soil N are also very important for increased maize production and productivity (Betrán et al., 2003a). Most often, drought, damage due to *Striga*, and soil nutrient deficiencies occur simultaneously in the field, and the combined effects can be disastrous (Cechin and Press, 1993; Kim et al., 1997). In a study conducted by Badu-Apraku et al. (2004) to examine the performance of cultivars with early maturity under induced moisture stress, *Striga* infestation, and optimal (well-watered and *Striga*-free) conditions, drought reduced grain yield by 53%, whereas 42% yield reduction resulted from *Striga* infestation. In a similar study, Badu-Apraku et al. (2010) demonstrated yield reductions of 44, 65, and 40% under drought, *Striga* infestation, and low N, respectively. In the northern Guinea and Sudan savannas where random drought stress is prevalent, it is important to introgress genes for drought tolerance into cultivars that possess resistance to *Striga*, since the two stresses occur simultaneously in the field. Badu-Apraku and Fakorede (2013) observed that maize farmers in the *Striga*-prone agroecologies of sub-Saharan Africa urgently need cultivars that are tolerant to drought and resistant to *Striga* and will not adopt maize cultivars that do not possess these characteristics. It is therefore important that maize breeding programs targeting the savannas of WCA pay special attention to at least drought stress and *Striga* infestation.

Maize breeding populations have been successfully improved for drought tolerance, yield, and other desirable agronomic traits through recurrent selection (Badu-Apraku et al., 1997; Chapman and Edmeades, 1999; Monneveux et al., 2006). For example, Edmeades et al. (1995) obtained yield gains per cycle of 175 kg ha^–1^ (14%) under drought, 162 kg ha^–1^ (3%) under optimal growing conditions, and 168 kg ha^–1^ (4.8%) across research environments in two early-maturing CIMMYT maize populations. Recurrent selection under induced drought stress at flowering and grain-filling periods resulted in annual yield gain of 5% under moisture stress (Edmeades et al., 1999).

Several researchers have shown that dominance and additive genetic effects were equally important in the inheritance of yield in temperate maize populations, whereas additive genetic effects were more important for other measured traits (Hallauer and Miranda, 1988; Han and Hallauer, 1989; Wolf et al., 2000). Similarly, Silva et al. (2004) investigated genetic variance in tropical maize populations and indicated that even though the magnitude of dominance and additive effects are specific for populations, they may differ depending on the dominance and additive gene action of the segregating loci. Nevertheless, there are few reports on the gene action modulating the inheritance of yield of tropical maize populations under drought stress, and they are also contradictory. For example, studies by Guei and Wassom (1992) revealed that the dominance genetic variance was greater than the additive variance for grain yield and ears per plant (EPP) in two tropical maize populations. In contrast, the authors showed that the additive genetic variance was more important than dominance variance in the expression of flowering traits under drought. Furthermore, Badu-Apraku et al. (2004) reported that additive genetic variance and narrow-sense heritability were moderate to large for yield and other measured traits in Pool 16 DT, after subjecting the population to eight selection cycles for increased yield under managed drought. They concluded that dominance genetic variance was equally important and needed to be considered in future selection programs.

Weyhrich et al. (1998) reported that the S_1_ progeny selection scheme has been designed to improve population performance and facilitate rapid fixation of alleles, with deleterious alleles exposed to the environment for elimination during the initial stages of the selection program. In the absence of over dominance, the S_1_ or S_2_ family selection method is superior to other population improvement methods (Lamkey, 1992). Weyhrich et al. (1998) investigated the responses to selection of a maize population using seven methods of recurrent selection and showed that all the methods were effective in improvement of population performance per se for yield. However, the greatest effect of selection for improved yield was observed using the S_2_ progeny selection method. They concluded that the selection method involving inbred progenies was responsible for the superior gains from selection in BS11 compared with other selection methods. Contrary to theoretical arguments that recurrent selection involving inbred progenies is superior to mass selection and the half-sib recurrent selection methods, it has been demonstrated empirically that S_1_ or S_2_ family selection is not always superior (Coors, 1999; Wardyn et al., 2009; Edwards, 2010). For example, Wardyn et al. (2009) demonstrated that predicted responses to S_1_ or S_2_ family selection did not show any advantages over half-sib methods in three maize populations. However, S_1_ or S_2_ family selection methods were unique and outstanding for improvement of inbred line performance, whereas selection methods involving half-sib progenies were superior for genetic enhancement of noninbred progenies. Wardyn et al. (2009) explained that linkage disequilibrium, overdominance, and/or epistasis could profoundly influence predictions from selection programs. Similarly, Edwards (2010) indicated that pseudo-overdominance because of linkage disequilibrium may limit responses to S_1_ or S_2_ family selection in maize breeding populations. Despite the limitations, the S_1_ recurrent selection method capitalizes on additive gene action and has been extensively used to screen segregating families of maize at IITA. Using this recurrent selection method, several early *Striga*-resistant or -tolerant cultivars with outstanding performance in drought-prone environments have been developed and released in sub-Saharan Africa. The early-maturing yellow maize population TZE-Y Pop DT STR with combined drought tolerance and *Striga* resistance was derived from diallel crosses involving elite maize germplasm identified and selected based on years of extensive multilocation testing in WCA. After the development of the population, the S_1_ family recurrent selection method has been used to improve it for *Striga* resistance and tolerance to drought and low N. The resulting improved population has been serving as a source population for extraction of outstanding maize products. Selection for early maturity has been conducted in the savanna and forest agroecologies of WCA, and several multiple-stress-tolerant cultivars have been developed. Several of these cultivars have been commercialized after wide testing in the subregion. Outstanding inbred lines selected for tolerance to drought and *Striga* resistance have been used for the development of early-maturing cultivars well adapted to drought stress and *Striga* endemic zones. The selected lines are also used as sources of beneficial alleles for improvement of early tropical breeding populations. Recurrent selection methods have been used to increase the frequency of beneficial genes to enhance tolerance to drought stress, whereas repeated self-pollination has also been used to fix desirable genes. A major goal of the breeding program has been the genetic enhancement of the breeding populations for *Striga* resistance and tolerance to water deficit. Using this strategy, TZE-Y Pop DT STR has been remarkably improved through the S_1_ recurrent selection program, aimed at concentrating beneficial genes in the breeding population. Through the recurrent selection and inbred–hybrid development programs, several inbred lines, open-pollinated cultivars, and hybrids with tolerance to multiple stress have been generated from the reference population (Badu-Apraku et al., 2006, 2008).

TZE-Y Pop STR was subjected to five S_1_ family recurrent selection cycles for improvement of *Striga* resistance level and increased grain yield performance under *Striga*-infested and noninfested environments (Badu-Apraku et al., 2007, 2008, 2009). After this was three selection cycles of improvement for tolerance to drought. Thus, the population possesses genes for combined resistance and tolerance to infestation by *Striga hermonthica* and drought stress. Selection and genetic drift in a target population may result in changes in gene frequencies and genetic variability. Consequently, after five selection cycles for improved yield and resistance to *Striga* and three cycles of selection for an upgraded level of drought tolerance in TZE-Y Pop DT STR, there is a need for information on changes in genetic parameters such as the genetic variability, heritability estimates, and genetic correlations due to recurrent selection in the population. Such information is highly desirable in determining the changes necessary in the population improvement methods and strategies to ensure continued gains from advanced selection cycles in the population enhancement program. Information on the genetic variance and heritability estimates in the target population for yield and other traits is crucial for ascertaining the effectiveness and progress anticipated from future selection cycles. It is therefore important to verify whether there existed sufficient genetic variability for yield and other desirable agronomic traits in the target population to facilitate future genetic gains from S_1_ progeny selection under drought stress, as well as to confirm the appropriateness of the breeding methodology adopted in the maize improvement program of IITA.

The present study was designed to (i) examine the genetic gains in yield and other desirable traits assayed during the three cycles of improvement in TZE-Y Pop DT STR under drought stress and optimal environments, (ii) estimate genetic variability and predict future gains from selection in the two research environments, and (iii) determine changes in relationships among the measured traits due to selection in the population under drought stress environments.

## MATERIALS AND METHODS

### Development of the Source Population and the S_1_ Recurrent Selection Program

An early-maturing, *Striga*-resistant, and drought-tolerant yellow source population, TZE-Y Pop DT C_0_ STR C_0_, was used for this study. The population was developed after recombination of the drought-tolerant yellow germplasm sources DR-Y Pool BC_2_F_2_, 9499, and KU 1414 using the half-sib method. The resulting early yellow population was named TZE-Y Pop. Subsequently, *Striga* resistance and tolerance genes from the IITA inbred line 9450 STR (Kim et al., 1987) were introgressed into TZE-Y Pop to improve the level of *Striga* resistance. This was followed by two cycles of backcrossing to the population. S_1_ progenies were then generated and outstanding *Striga*-resistant S_1_ lines were selected and subjected to two cycles of random mating under artificial *Striga* infestation and induced moisture stress to form TZE-Y Pop DTC_0_ STR C_0_. The methodologies and strategies used for evaluation for resistance to *Striga* and the induced moisture stress management practices of selection for tolerance to drought at different stages during the development of the population at various screening sites in WCA were fully described by Badu-Apraku et al. (2007). In brief, the S_1_ family selection scheme was initiated in the population in 1996 and has gone through five cycles of selection for improved yield and *Striga* resistance, followed by three selection cycles for enhanced tolerance to drought. Progenies derived from cycles of genetic improvement were evaluated under artificial *S. hermonthica* infestation and noninfested conditions from 1996 to 2001 at Ferkéssedougou in Côte d’Ivoire and Abuja and Mokwa in Nigeria in 2003. Per cycle, 196 to 256 progenies were screened, using a selection intensity of 25 to 30%. Based on the across-location data, 25 to 30% best S_1_ progenies derived from the population were identified using the IITA base index that incorporated grain yield, *Striga* emergence counts, *Striga* damage rating at 8 and 10 wk after planting, and EPP assayed under *Striga* infestation and/or no *Striga* infestation (MIP, 1996). The selected ears of the best S_1_ families of each cycle of the population were intermated to form a new, enhanced population for the new cycle of selection. A minimum of three seasons were needed to complete a cycle of selection. By 2007, five cycles of selection for *Striga* resistance had been completed. The initial phase of the genetic enhancement for tolerance to drought was performed under managed drought stress at Ferkéssedougou and Sinématialli in Côte d’Ivoire and Kamboinse in Burkina Faso (Badu-Apraku et al., 2008). Subsequently, drought screenings were conducted at Ikenne and Bagauda in Nigeria. The details on the improvement program were described by Badu-Apraku et al. (2012, 2015). In brief, at Sinématialli, Ferkéssedougou, and Ikenne, the drought experiments were performed using an overhead irrigation system that supplied 17 mm water wk^–1^ during the dry season. The managed drought stress at Ferkéssedougou and Sinématialli was obtained by withdrawing irrigation water from ~2 wk before anthesis to the end of the growing season. In contrast, at Ikenne, planting was done during the dry season. Managed drought was obtained through suspension of the irrigation of the plants in the plots at 28 d after planting until physiological maturity, thus compelling the plants to rely on stored water in the soil for growth and development. The soil at the testing site in Ikenne is characterized as an Alfisol, with experimental fields that are flat and uniform and have high water-holding capacity (Soil Survey Staff, 1999). At Bagauda, plants were exposed to natural terminal drought, which normally coincides with the flowering period and continues until harvest maturity. The optimal experiments were performed at Ikenne during the major growing season (rainfed). With the exception of the amount of water applied in the optimal environments, all management practices were similar for both optimal and drought experiments. The application of fertilizer and weed control in the optimal and drought-stressed plots was as described by Badu-Apraku et al. (2015). The best S_1_ lines under drought stress were selected using the base index described by Meseka et al. (2006) and Oyekunle and Badu-Apraku (2012). A minimum of three seasons were necessary for completing a selection cycle for drought tolerance. The population had been subjected to five selection cycles for enhanced grain yield and *Striga* resistance, as well as three cycles of genetic enhancement for grain yield and drought tolerance, when the present experiment was commenced in 2011. However, at C_2_ of the population, a study was conducted to determine the genetic variability of grain yield and other assayed traits under drought. Results of this study revealed low genetic variability for most measured traits, and a recommendation was made for incorporation of new sources of drought tolerance genes into the population to accelerate progress from further selections in the population. Consequently, genes for drought tolerance were introgressed from the panel of drought-tolerant inbreds from IITA and CIMMYT into the population. The introgression of drought tolerance genes was followed by a cycle of selection and recombination to form the C_3_ of the population.

### Field Evaluations and Crop Management

We evaluated 60 S_1_ families each derived by self-pollination of noninbred plants from the *Striga*-resistant population TZE-W Pop DT C_0_ STR C_5_ and the three cycles of selection for improved drought tolerance under drought stress (i.e., C_0_ to C_3_). The 240 S_1_ families from C_0_ to C_3_ were tested under managed drought stress at Ikenne during the dry seasons of 2010–2011 and 2011–2012 and optimal environments at Ikenne in 2011 and 2012 and Kadawa during the rainy season of 2011. Badu-Apraku et al. (2012, 2015) provided a complete description of the drought methodology adopted for evaluation of the cycles of selection in the present study. A 15 × 16 lattice with two replicates was used for the field evaluations. Single-row plots, each measuring 3 m with a row spacing of 0.75 m and a distance of 0.4 m between plants within rows, were used. Three seeds were planted per hole, and the seedlings were thinned to two per stand ~2 wk after emergence to obtain a final plant population density of ~66,667 plants ha^–1^. Application of fertilizer and weed management practices in the optimal and induced drought stress plots were performed at Ikenne and Kadawa, following methods described by Badu-Apraku et al. (2015).

### Data Collection

Recorded data in both induced drought stress and optimal (rainfed or well-watered) plots were as detailed by Badu-Apraku et al. (2015) for days to 50% anthesis (DA) and silking (DS), anthesis–silking interval (ASI), plant height (PHT), ear height (EHT), root lodging, stalk lodging, stay-green characteristic (STGR), husk cover (HUSK), ear aspect (EASP), plant aspect (PASP), and EPP. For trials under both drought and optimal environments, grain moisture was determined from shelled kernels from each plot. Grain yield for drought trials was estimated from the shelled grain weight, adjusted to 15% moisture content. However, for well-watered environments, grain weight was estimated from cob weight, assuming 80% shelling percentage, adjusted to 15% moisture.

### Statistical Analyses

The plot means of the individual traits combined for the locations-within-year were subjected to ANOVA using the PROC GLM command of SAS 9.3 (SAS Institute, 2011). In the combined ANOVA for each trait, the environments comprised the location-within-year combinations. In the model, the effects of environment, replication, incomplete blocks, and the interactions of environment with cycle and genotype-within-cycle were assumed as random effects. In contrast, cycle and genotype-within-cycle effects were regarded as fixed effects in the computation of the means and SEs per cycle, whereas they were considered as random effects for the estimation of variance components. The analyses were performed separately for the managed drought and optimal growing conditions.

The estimates of genetic variance for assayed traits were obtained by equating the observed to the expected mean squares and calculating the desired components as proposed by Hallauer et al. (2010). The incomplete block effect of the model was disregarded, and the error variance was estimated. Standard errors for genetic variance and heritability estimates were computed based on the method proposed by Hallauer et al. (2010). The estimates of genetic variance and heritability among S_1_ families of the different cycles were compared for differences by a pairwise test of estimates using the SEs. The predicted selection gain (*G*_s_) was based on C_3_ alone and was determined using the method of Hallauer et al. (2010) as follows:

$$G_{s}=k\sigma_{g}^{2}/\sigma_{p}$$

where *k* is the standardized selection differential for S_1_ families (based on 20% selection pressure, *k* = 1.3998), $\sigma_{g}^{2}$ is the genetic variance, and σ_p_ represents the square root of the phenotypic variance. The predicted selection gains may have been inflated based on the proportion contributed by nonadditive genetic variance to the genetic variance. Edwards (2008) proposed a more accurate method for predicting gains from selection, which involves the use of additive variance instead of genetic variance as the numerator. This method provides more accurate predictions of gain from selection with inbred progeny than the method proposed by Hallauer et al. (2010). However, the method is more complex, and the computation is more challenging and was therefore not adopted in the present study. The differences among the cycle means were tested for significance using the LSD. Linear contrast was performed using SAS 9.3 (SAS Institute, 2011) to partition the cycle mean squares of measured traits into single degrees of freedom for orthogonal comparisons, which involved C_0_ vs. C_1_ + C_2_ + C_3_, C_1_ vs. C_2_ + C_3_, and C_2_ vs. C_3_ for yield and most of the other assayed traits under drought stress and optimal growing environments. The *b* values obtained from regression of the measured trait on the selection cycles provided an estimate of realized gain per cycle, whereas the percentage response per cycle was estimated as (realized gain cycle^–1^/intercept) × 100. The significance of the slope *b* was tested using the *t* test at 0.05 probability level.

We used SPSS version 17.0 (SPSS, 2007) to carry out the stepwise regression analyses on the assayed traits. Subsequently, sequential path diagrams were used to explain the cause-and-effect relationships among traits in the cycles C_0_ and C_3_ of selection for tolerance to drought as described by Mohammadi et al. (2003). Badu-Apraku et al. (2012, 2014) and Talabi et al. (2017) described in detail the procedures adopted for the sequential stepwise multiple regression analysis in the present study.

## RESULTS

### Analysis of Variance and Progress from Selection under Different Environments

Combined ANOVA revealed significant cycle effects for yield and other traits assayed under drought stress and optimal environments (Table 1). Significant environmental effects were obtained for measured traits except for yield, ASI, PASP, and EPP under drought stress and DS under optimal environments. The genotype × environment interaction (GEI) mean squares were significant for yield, PHT, PASP, and EASP under drought stress and optimal environments. A significant GEI was also observed for STGR under drought. Under drought, mean yield varied from 803 kg ha^–1^ for C_2_ to 2384 kg ha^–1^ for C_3_, and under optimal environments, from 1831 kg ha^–1^ for C_2_ to 3737 kg ha^–1^ for C_3_ (Table 1). The most advanced cycle of selection, C_3_, significantly (*P* < 0.05) yielded higher than the preceding cycles of selection under the two research conditions (drought stress and optimal growing environments). In addition to the higher grain yield in C_3_, there were decreased DS and ASI, improved PASP and EASP, and increased PHT and EPP under drought stress and optimal growing environments. Stay-green characteristic of C_3_ under drought was also better than in the other selection cycles. The yield gain per cycle from C_0_ to C_3_ was 423 kg ha^–1^ (202%) under drought and 518 kg ha^–1^ (48%) under optimal environments. The predicted selection gain per cycle was 348 kg ha^–1^ for yield under drought and 377 kg ha^–1^ under optimal conditions. The mean squares from linear contrast of cycles revealed significant effects for the comparisons of C_0_ vs. C_1_ + C_2_ + C_3_, C_1_ vs. C_2_ + C_3_, and C_2_ vs. C_3_ for yield and most other traits assayed under drought and optimal environments (Table 2). The few exceptions included the contrasts C_0_ vs. C_1_ + C_2_ + C_3_ for ASI under optimal environment, and C_1_ vs. C_2_ + C_3_ for ASI and STGR under drought. The contrast C_2_ vs. C_3_ accounted for 74 and 72% of the total cycle mean squares for yield under drought and optimal environments, respectively. Similarly, the contrast C_2_ vs. C_3_ contributed >70% to the total cycle effects of other traits assayed under drought and optimal environments, except for DS and ASI under optimal conditions.

**Table 1:** Response per cycle, grain yield, and other traits of S_1_ families derived from four cycles of selection in an early yellow population evaluated under drought (DST) at Ikenne during the 2010–2011 and 2011–2012 dry seasons and under optimal conditions (WW) at Ikenne during the 2011 and 2012 growing seasons and at Kadawa during the 2011 growing season in Nigeria.

Cycle	Grain yield		Days to Silking		Anthesis-silking interval		Plant height		Plant aspect		Ear aspect		Stay-green characteristic		Ears per plant	
	DST	WW	DST	WW	DST	WW	DST	WW	DST	WW	DST	WW	DST	DST	WW
	— kg ha^–1^ —		——————— d ———————				—— cm ——								
C_0_†	918	1993	60	57	5.0	1.2	158.1	167.8	3.4	3.2	3.5	3.2	4.5	0.52	0.83
C_1_	968	1887	60	58	4.8	1.5	157.8	166.4	3.3	3.2	3.4	3.3	4.4	0.54	0.82
C_2_	803	1831	61	57	5.6	1.4	148.3	161.8	3.5	3.3	3.5	3.3	4.5	0.50	0.81
C_3_	2384	3737	56	55	3.5	0.8	181.5	185.0	2.7	2.2	2.7	2.5	4.1	0.77	0.97
Grand mean	1173	2457	60	57	4.7	1.1	156.7	170.1	3.3	3.0	3.3	3.0	4.3	0.55	0.88
LSD	104.9	117.3	0.6	0.4	0.39	0.16	3.62	2.40	0.08	0.07	0.07	0.06	0.11	0.032	0.030
Environment (E)	ns‡	**	**	ns	ns	**	*	**	ns	**	*	**	**	ns	**
Cycle (G)	**	**	**	**	**	**	**	**	**	**	**	**	**	**	**
G × E	**	**	ns	ns	ns	ns	**	**	**	**	**	**	**	ns	ns
Realized gain	423	518	–1.21	–0.94	–0.39	–0.15	6.06	4.68	–0.21	–0.30	–0.20	–0.21	–0.08	0.07	0.04
Response cycle^–1^ (%)	202	48	–1.93	–1.58	–6.67	–9.49	4.14	2.95	–5.61	–8.06	–5.28	–5.84	–1.83	18	5.39
Predicted gain cycle^–1^ (based on C_3_)	348	377	1.0	0.76	0.39	0.08	0	1.42	0.08	0	0.17	0.11	0	0.08	0

Significant at the 0,05 and 0.01 probability levels, respectively.

† C_0_, C_1_, C_2_, and C_3_ referto the base population, Cycle 1, Cycle 2, and Cycle 3, respectively.

‡ ns, nonsignificant.

**Table 2:** Sums of squares from linear contrast of grain yield and other traits of S1 families derived from four cycles of selection in an early yellow population evaluated under drought (DST) at Ikenne during the 2010–2011 and 2011–2012 dry seasons and under optimal conditions (WW) at Ikenne during the 2011 and 2012 growing seasons and at Kadawa during the 2011 growing season in Nigeria.

Cycle	df	Grain yield		Days to Silking		Anthesis-silking interval		Plant height		Plant aspect		Ear aspect		Stay-green characteristic		Ears per plant	
		DST	WW	DST	WW	DST	WW	DST	WW	DST	WW	DST	WW	DST	DST	WW
C_0_† vs. C_1_ + C_2_ + C_3_	1	36,889,190**	70,337,674**	305**	183**	21*	0.2ns‡	2,239*	3,317**	7**	22**	9**	11**	3.00**	1.18**	0.43**
C_1_ vs. C_2_ + C_3_	1	59,502,597**	175,112,390**	499**	1,148**	11ns	43.1**	7,265**	8,640**	11**	53**	14**	33**	0.01ns	1.61**	0.96**
C_2_ vs. C_3_	1	280,545,770**	626,682,342**	1,988**	969**	478**	49.3**	107,877**	87,793**	73**	223**	65**	109**	14.97**	8.38**	4.74**
	**Contribution of linear contrasts to the total sums of squares**															
	—————————————————————————————————— % ————————————————————————————————															
C_0_ vs. C_1_ + C_2_ + C_3_	1	9.79	8.07	10.92	7.96	4.12	0.22	1.91	3.33	7.69	7.38	10.23	7.19	16.69	10.56	7.01
C_1_ vs. C_2_ + C_3_	1	15.79	20.08	17.87	49.91	2.16	46.54	6.19	8.66	12.09	17.79	15.91	21.57	0.06	14.41	15.66
C_2_ vs. C_3_	1	74.43	71.86	71.20	42.13	93.73	53.24	91.90	88.01	80.22	74.83	73.86	71.24	83.26	75.02	77.32

*,** Significant at the 0,05 and 0,01 probability levels, respectively.

† C_0_, C_1_, C_2_, and C_3_ referto the base population, Cycle 1, Cycle 2, and Cycle 3, respectively.

‡ ns, nonsignificant.

### Genetic Variance and Broad-Sense Heritability Estimates

Under drought, genetic variance estimates showed significant effects for yield, DA, EHT, and EPP in C_0_; grain yield, DA, DS, PHT, EHT, HUSK, and EPP in C_1_; DA, ASI, EHT, and EPP in C_2_; and grain yield, EASP, and EPP in C_3_ (Table 3). The heritability estimates followed similar trends. Under optimal environments, significant genetic variance and heritability estimates were obtained for all traits assayed in cycles C_0_, C_1_, C_2_, and C_3_ except EPP in C_0_, PASP in C_1_, grain yield, ASI, PASP, EASP, and EPP in C_2_, and ASI, PHT, EHT, PASP, and EPP in C_3_ (Table 4).

**Table 3:** Genetic variance and heritability estimates of S_1_ families derived from four cycles of selection in an early yellow population evaluated under managed drought at Ikenne during the 2010–2011 and 2011–2012 dry seasons in Nigeria.

Trait	Genetic variance				Heritability			
	C_0_†	C_1_	C_2_	C_3_	C_0_	C_1_	C_2_	C_3_
Grain yield	50,858 ± 20,488*	89,438 ± 26,423**	23,048 ± 17,674	133,219 ± 59,021*	0.50 ± 0.20*	0.65 ± 0.19*	0.29 ± 0.22	0.46 ± 0.21*
Days to anthesis	1.38 ± 0.57*	1.45 ± 0.53*	1.69 ± 0.61*	0.40 ± 0.31	0.49 ± 0.20*	0.55 ± 0.20*	0.55 ± 0.20*	0.28 ± 0.22
Days to silking	1.26 ± 1.05	2.88 ± 1.18*	2.10 ± 1.18	1.28 ± 0.68	0.27 ± 0.22	0.50 ± 0.20*	0.38 ± 0.21	0.40 ± 0.21
Anthesls-sllklng Interval	0.73 ± 0.41	0.56 ± 0.40	1.69 ± 0.62*	0.26 ± 0.19	0.38 ± 0.21	0.31 ± 0.22	0.55 ± 0.20*	0.30 ± 0.22
Plant height	72 ± 40	73 ± 29*	24 ± 28	0 ± 29	0.38 ± 0.21	0.50 ± 0.20*	0.19 ± 0.23	0.00 ± 0.28
Ear height	45 ± 16*	38 ± 18*	33 ± 16*	11 ± 16	0.54 ± 0.20*	0.44 ± 0.21*	0.42 ± 0.21*	0.17 ± 0.24
Husk cover	0.014 ± 0.016	0.039 ± 0.018*	0.009 ± 0.017	0.009 ± 0.010	0.20 ± 0.23	0.44 ± 0.21*	0.13 ± 0.24	0.20 ± 0.23
Plant aspect	0.0049 ± 0.018	0.0202 ± 0.017	0.0002 ± 0.018	0.0153 ± 0.015	0.07 ± 0.25	0.27 ± 0.22	0.00 ± 0.26	0.23 ± 0.23
Ear aspect	0.011 ± 0.011	0.029 ± 0.011*	0.003 ± 0.012	0.034 ± 0.016*	0.24 ± 0.23	0.54 ± 0.20*	0.06 ± 0.25	0.44 ± 0.21*
Ears per plant	0.006 ± 0.003*	0.014 ± 0.004**	0.005 ± 0.002*	0.006 ± 0.003*	0.44 ± 0.21*	0.68 ± 0.19**	0.43 ± 0.21*	0.54 ± 0.20*
Stay-green characteristic	0.029 ± 0.023	0.000 ± 0.032	0.050 ± 0.039	0.000 ± 0.018	0.28 ± 0.22	0.00 ± 0.26	0.29 ± 0.22	0.00 ± 0.29

Significant at the 0,05 and 0.01 probability levels, respectively.

† C_0_, C_1_, C_2_, and C_3_ referto the base population, Cycle 1, Cycle 2, and Cycle 3, respectively.

**Table 4:** Genetic variance and heritability estimates of S_1_ families derived from four cycles of selection in an early yellow population evaluated under optimal conditions at Ikenne during the 2011 and 2012 growing seasons and at Kadawa during the 2011 growing season in Nigeria.

Trait	Genetic variance				Heritability			
	C_0_†	C_1_	C_2_	C_3_	C_0_	C_1_	C_2_	C_3_
Grain yield	90,581 ± 35,298*	99,918 ± 43,546*	57,035 ± 35,232	138,=502 ± 26,055**	0.49 ± 0.19*	0.45 ± 0.19*	0.33 ± 0.20	0.52 ± 0.17**
Days to anthesis	1.65 ± 0.47**	1.79 ± 0.53**	1.27 ± 0.39**	0.41 ± 0.09**	0.65 ± 0.19**	0.63 ± 0.19**	0.61 ± 0.19**	0.49 ± 0.08**
Days to silking	2.42 ± 0.67**	2.02 ± 0.63**	1.79 ± 0.56**	0.59 ± 0.13**	0.67 ± 0.19**	0.60 ± 0.19**	0.60 ± 0.19**	0.50 ± 0.09**
Anthesis-silking interval	0.25 ± 0.08**	0.19 ± 0.09*	0.07 ± 0.07	0.02 ± 0.02	0.57 ± 0.19**	0.44 ± 0.19*	0.21 ± 0.21	0.16 ± 0.19
Plant height	83 ± 23**	60 ± 20**	47 ± 19*	7 ± 7	0.68 ± 0.19**	0.58 ± 0.19**	0.48 ± 0.19*	0.16 ± 0.16
Ear height	30 ± 10**	20 ± 9*	18 ± 9*	5 ± 5	0.57 ± 0.19**	0.43 ± 0.20*	0.39 ± 0.20	0.18 ± 0.19
Husk cover	0.058 ± 0.020*	0.051 ± 0.019*	0.069 ± 0.027*	0.028 ± 0.009**	0.56 ± 0.19*	0.51 ± 0.19*	0.49 ± 0.19*	0.38 ± 0.10**
Plant aspect	0.0325 ± 0.0145*	0.0093 ± 0.0112	0.0280 ± 0.0176	0 ± 0.0104	0.44 ± 0.20*	0.17 ± 0.21	0.32 ± 0.20	0 ± 0.22
Ear aspect	0.018 ± 0.007*	0.030 ± 0.010**	0.018 ± 0.010	0.018 ± 0.007*	0.49 ± 0.19*	0.57 ± 0.19**	0.35 ± 0.20	0.33 ± 0.12**
Ears per plant	0.003 ± 0.002	0.007 ± 0.003*	0.003 ± 0.003	0.000 ± 0.001	0.34 ± 0.20	0.41 ± 0.20*	0.24 ± 0.21	0 ± 0.15

Significant at the 0.05 and 0.01 probability levels, respectively.

† C_0_, C_1_, C_2_, and C_3_ refer to the base population, Cycle 1, Cycle 2, and Cycle 3, respectively.

### Stepwise Multiple Regression and Sequential Path Analyses

**Fig. 1:**
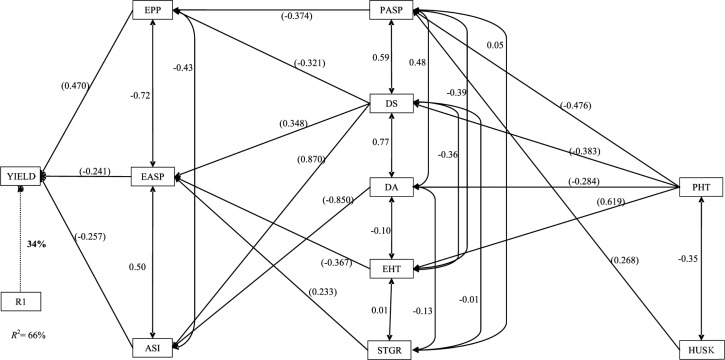
Path analysis model diagram showing causal relationships of measured traits of early-maturing S_1_ lines developed from cycle C_0_ of TZE-Y Pop DT STR evaluated under drought stress at Ikenne during the 2010–2011 and 2011–2012 dry seasons in Nigeria. The bold value is the residual effect. Values in parentheses are direct path coefficients, whereas other values are correlation coefficients. R1, residual effects; ASI, anthesis–silking interval; DA, days to 50% anthesis; DS, days to 50% silking; EASP, ear aspect; EPP, ears per plant; HUSK, husk cover; PASP, plant aspect; PHT, plant height; STGR, stay green characteristics; YIELD, grain yield.

The EPP, ASI, and EASP were identified by the stepwise multiple regression analysis as traits with significant contributions to yield, accounting for ~66% of the total variation in yield among the S_1_ lines developed from the base population (cycle C_0_) of TZE-Y Pop DT STR and evaluated under drought (Fig. 1). Among these three traits, EPP had the greatest and only positive direct effect (0.470) on yield, whereas the effects of the other two traits were negative and much smaller (Fig. 1). Indirect contributions were made by several other traits to yield through one or more of the first-order traits. Among the five traits (PASP, DS, DA, EHT, and STGR) in the second order, DS indirectly contributed to yield through all the three primary traits, whereas the others indirectly contributed to yield through only one of the first-order traits. The greatest positive indirect effect (0.870) was made by DS through ASI, whereas the greatest negative indirect effect (–0.850) was contributed by DA through ASI. The remaining indirect contributions of the three second-order traits to yield through the first-order traits are illustrated in Fig. 1. Only two measured traits, PHT and HUSK, were identified as the third-order traits with significant indirect effects on yield. Although PHT made indirect contributions through four of the second-order traits, the HUSK made indirect contribution through only one trait, PASP (0.268).

**Fig. 2:**
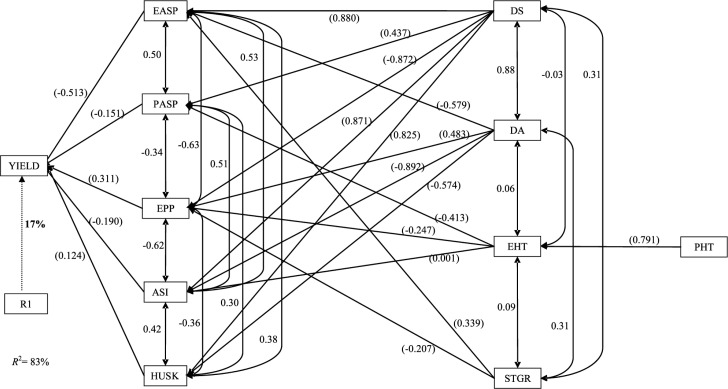
Path analysis model diagram showing causal relationships of measured traits of early-maturing S_1_ lines developed from cycle C_3_ of TZE-Y Pop DT STR and evaluated under drought stress at Ikenne during the 2010–2011 and 2011–2012 dry seasons in Nigeria. The bold value is the residual effect. Values in parentheses are direct path coefficients, whereas other values are correlation coefficients. R_1_, residual effects; ASI, anthesis–silking interval; DA, days to 50% anthesis; DS, days to 50% silking; EASP, ear aspect; EPP, ears per plant; HUSK, husk cover; PASP, plant aspect; PHT, plant height; STGR, stay green characteristic; YIELD, grain yield.

For the S_1_ lines developed from the cycle C_3_ of the TZE-Y Pop DT STR population and tested under drought stress, five traits including EASP, PASP, EPP, ASI, and HUSK were identified as the first-order contributors to yield. About 83% of the total variation in grain yield was attributable to these traits (Fig. 2). Ear aspect made the greatest direct contribution to yield (–0.513), whereas only EPP (0.311) and HUSK (0.124) had positive direct effects on yield. Only four traits, DS, DA, EHT, and STGR, were categorized into the second-order group, and these made contributions through five, four, three, and two second-order traits, respectively. Out of the 14 indirect contributions of the second-order traits, seven effects were positive values, three of which had indirect path coefficients >0.800 (Fig. 2). Plant height was identified as the only third-order trait that contributed through EHT (0.791) to grain yield.

## DISCUSSION

The observed significant differences (*P* < 0.01) among the four cycles of S_1_ families for yield and all other traits assayed under drought and optimal growing environments indicated that genetic variability existed in the early-maturing yellow breeding population TZE- Y Pop DT STR studied and that genetic gains could be achieved for the original cycle and the subsequent recurrent selection cycles (Badu-Apraku et al., 2011, 2016; Berilli et al., 2013). The highly significant environmental effects (*P* < 0.01) for all traits assayed except for yield, ASI, PASP, and EPP under drought and DS, ASI, and EPP under optimal conditions indicated that the research environments in Nigeria varied in terms of climatic and edaphic conditions. Furthermore, the presence of significant GEI means squares for most of the measured traits under drought stress and optimal growing environments suggested that there were differences in the responses of the S_1_ lines in the different cycles to environmental variations, and that the research environments were discriminating enough in the identification of outstanding cultivars in the recurrent selection procedure (Badu-Apraku et al., 2016). The observed significant GEI for grain yield, PHT, PASP, and EASP under both research conditions and the STGR under drought may be due largely to differences in environmental factors, particularly soil type, temperature, amount of rainfall, and disease pressure at the testing sites in Nigeria. This is also an indication of the uniqueness of the environments in the identification of outstanding cultivars. Furthermore, the significant GEI effects for yield and most other traits in the base index for identification of tolerance to drought indicated that the genotypic correlation of these traits with yield was expected to decrease under drought stress. In contrast, the nonsignificant GEI for DS, ASI, and EPP under both drought and optimal conditions suggested that there would be consistency in the expression of these traits in the contrasting environments (Badu-Apraku et al., 2016).

The mean of a trait is a parameter of paramount importance in population improvement because a high population mean indicates that a shorter time is required to achieve the targeted level of progress, and vice versa (Hallauer et al., 2010). The relatively high cycle mean grain yields recorded for C_3_, as well as the significant yield gains achieved from C_0_ to C_3_ under drought stress and optimal growing environments, confirmed the significant improvements in yield achieved in the advanced cycles of selection. This result therefore suggested that a relatively short period would be required to achieve substantial progress in advanced cycles. It was also striking that the relatively high mean yield obtained for C_3_ was accompanied by decreased silking dates, reduced ASI, improved PASP and EASP, and increased PHT and EPP under drought and optimal environments, as well as improved STGR under drought. Anthesis–silking interval, STGR, PASP, EASP, and EPP are secondary traits included in the IITA selection index for identification of superior cultivars under drought conditions (Meseka et al., 2006; Badu-Apraku et al., 2013). Edmeades et al. (1996) and Bänziger et al. (2000) demonstrated that selection efficiency for tolerance to drought could be improved through utilization of secondary traits that could be easily measured, have high heritability, and are strongly correlated with yield under stress. To achieve significant genetic enhancement for improved yield under drought, Bänziger and Lafitte (1997) combined information from selected secondary traits in a Smith–Hazel index and obtained an average of 14% improved selection efficiency compared with selection for yield alone. A similar base index is also used by IITA scientists to select for drought tolerance under drought and optimal growing environments (Badu-Apraku et al., 2013).

The yield gain per cycle from C_0_ to C_3_ was very high (202%) under drought and moderately high (48%) under optimal conditions. This suggested that there was high frequency of favorable drought tolerance alleles in the early yellow population for continued progress from future recurrent selection programs, as additional cycles of recombination took place (Halward and Wynne, 1992). Furthermore, results of the present study revealed that predicted gain per cycle for grain yield (348 kg) under drought was smaller than that under optimal conditions (377 kg). This result is contrary to the findings of Badu-Apraku et al. (2016), who studied the *Striga*-resistant and drought-tolerant early-maturing white population TZE-W Pop DT C_3_ STR C_5_ under drought stress and well-watered environments. The authors reported that the genetic variance generally decreased for yield and other traits assayed in advanced cycles of the population under drought and well-watered conditions, except for yield and EHT under well-watered conditions. Similarly, heritability estimates for yield and other measured traits decreased in the advanced cycles of selection in the population under drought but increased in advanced cycles under well-watered conditions. Realized gain from selection for yield was 0.291 t ha^–1^, corresponding to 30.5% cycle^–1^ under drought and 0.352 t ha^–1^ with a corresponding gain of 16.7% cycle^–1^ under well-watered conditions. Predicted selection gain based on C_3_ was 0.282 and 0.583 t ha^–1^ under drought and well-watered conditions. Low estimates of genetic variance, heritability, and predicted gains from selection for yield and other traits suggested the need to introgress drought tolerance genes into the TZE-W Pop DT C_3_ STR C_5_ population (Badu-Apraku et al., 2016). A plausible reason for the contrasting results of the two studies is that the two populations were derived from different sources of germplasm and might have varied in the mechanisms by which they achieved drought tolerance.

It is not surprising that higher predicted gain per selection cycle was obtained under drought stress than under optimal growing conditions because the recurrent selection scheme placed greater emphasis on grain yield performance under drought than under optimal conditions. This could also have been due to biased estimation of the predicted gain per cycle at C_3_ under optimal conditions, as the yield gain was ~36.2% higher than that under drought conditions.

In a recurrent selection program, estimating heritability for a trait from the genetic variance components is useful to determine the amount of progress that could be made in the improvement of that trait. Genetic variance, which is directly related to heritability estimates, initially increased for yield and other traits assayed from C_0_ to C_1_ but declined from C_1_ to C_2_ and subsequently showed a marked increase from C_2_ to C_3_. Although this result showed inconsistent patterns in genetic variance as selection progressed under drought and optimal conditions, it is interesting that the greatest progress made was from C_2_ to C_3_ which was the most advanced cycle of selection for drought tolerance in the present study. This progress is not surprising and could be attributed to the introgression of beneficial alleles for drought tolerance from selected drought-tolerant inbred lines from IITA and CIMMYT drought-tolerant inbreds into the population at C_2_, followed by a selection cycle and recombination to reconstitute the C_3_ of the population. Significant linear contrast observed for the orthogonal comparisons indicated that there were wide differences among the contrasts. However, >70% of the total cycle mean squares attributable to the comparison between C_2_ and C_3_ indicated that gains from selection resulted from the introgression of beneficial alleles for drought tolerance followed by one selection cycle and recombination. The high genetic variance observed for grain yield in the C_3_ under both research conditions indicated that there was probably genetic variability for most of the traits assayed to facilitate the response to continued selection for improvement of yield in the population. This might have resulted from the selection of desirable genes for both tolerance to drought and improved performance under optimal conditions at particular loci of the S_1_ lines as influenced by factors such as the recombination rate, selection intensity, mutation rate, genetic drift, the mating systems, population structure, and genetic linkage (Hallauer and Miranda, 1988). In addition, the high genetic variation for grain yield in the C_3_ correlated with a moderately high broad-sense heritability estimate compared with C_2_. This observation was anticipated, as it justified the fact that heritability is the fraction of the variance of a trait within a population that is due to genetic factors. The moderately high heritability estimates recorded for yield, DS, EPP, and EASP under drought in C_3_ indicated higher probability of improving the early yellow population for these traits in subsequent cycles of the recurrent selection program. The reduction in the genetic variance of S_1_ lines in C_2_ might have occurred due to a more intense selection pressure imposed on the measured traits at the C_1_ to C_2_ stage.

Path coefficient analysis facilitates the examination of the magnitude of varying contribution of different agronomic traits to grain yield in the form of cause and effect (Wright, 1921, 1923; Dewey and Lu, 1959). It describes the effective measurement of direct and indirect causes of association and illustrates the relative importance of each factor contributing to the final product (i.e., grain yield). Under drought, EPP, EASP, and ASI were identified as the first-order traits assayed with significant direct effects on grain yield of the S_1_ progenies, thus suggesting that these traits played key roles in the improvement of yield under moisture stress conditions. In addition, this result did not only show that EPP, EASP, and ASI were important secondary traits contributing to drought tolerance (Badu-Apraku et al., 2011, 2013; Talabi et al., 2017), but it also revealed that the progress from selection under drought using grain yield along with the secondary traits in a selection index would be greater than selecting for grain yield alone (Bänziger et al., 2000; Badu-Apraku et al., 2013). Therefore, it may be concluded that there was effective direct selection for these traits in C_0_. Furthermore, among the second-order traits, DS made significant indirect contributions to yield through the three first-order traits and made the highest positive indirect contribution to grain yield through ASI. Days to 50% silking also showed a high positive association with DA among the second-order traits, with the two traits having direct effects on ASI in C_0_ under drought, accounting for the observed variation in grain yield. Several workers (Bänziger and Lafitte, 1997; Betrán et al., 2003b; Badu-Apraku et al., 2011, 2013; Talabi et al., 2017) have reported that reduced ASI contributed to increased yield under drought stress.

Under drought stress, the sequential path coefficient analysis was very effective in providing insight into the variations observed in grain yield of the S_1_ families at C_3_, as indicated by the *r*^2^ of 83%. Among the five first-order traits (EASP, PASP, EPP, ASI, and HUSK) identified as having direct effects on grain yield, three of them (ASI, EPP, and EASP) were also categorized as the first-order traits with direct contribution to grain yield at C_0_, indicating the consistency and the importance of these three traits as selection progressed from C_0_ to C_3_. Similarly, the C_0_ and C_3_ S_1_ lines evaluated under drought conditions identified common second-order traits, except PASP at C_0_, in their indirect contributions to grain yield. The obvious direct contributions of DS and DA to reduced ASI, as well as the direct effects of STGR on both EASP and EPP, indirectly accounted for the superior yield performance of the C_3_ S_1_ lines in water-deficit environments.

## CONCLUSIONS

The large realized genetic gains from selection for improved yield under drought and optimal growing environments could be attributed to the introgression of favorable genes for drought tolerance into the C_2_ of the population, followed by one S_1_ family cycle selection and recombination. Furthermore, high genetic variability, heritability, and predicted gains for yield and other assayed traits in the most advanced cycle of improvement in the population indicated that further improvement of such traits is possible in future selection cycles in the population, depending largely on the ability of the breeders to identify outstanding genotypes and the precision levels of the experiments. In addition, EPP, EASP, ASI, PASP, and HUSK contributed directly to high grain yields under drought conditions both in C_0_ and C_3_, confirming the high reliability of the traits for effective selection for improved yield under drought stress. The ASI, EPP, and EASP were consistent and reliable secondary traits under drought as selection progressed from C_0_ to C_3_, confirming their effectiveness for index selection for drought tolerance.

## Abbreviations

ASI,: anthesis–silking interval;
DS,: days to 50% silking;
DA,: days to 50% anthesis;
EASP,: ear aspect;
EHT,: ear height;
EPP,: ear number per plant;
GEI,: genotype × environment interaction;
HUSK,: husk cover;
PASP,: plant aspect;
PHT,: plant height;
STGR,: stay-green characteristic;
WCA,: West and Central Africa.
